# Response of the human myocardium to ischemic injury and preconditioning: The role of cardiac and comorbid conditions, medical treatment, and basal redox status

**DOI:** 10.1371/journal.pone.0174588

**Published:** 2017-04-05

**Authors:** Kelly Casós, Gemma Ferrer-Curriu, Paula Soler-Ferrer, María L Pérez, Eduard Permanyer, Arnau Blasco-Lucas, Juan Manuel Gracia-Baena, Miguel A Castro, Carlos Sureda, Jordi Barquinero, Manuel Galiñanes

**Affiliations:** 1 Reparative Therapy of the Heart, Vall d’Hebron Research Institute (VHIR), University Hospital Vall d’Hebron, Autonomous University of Barcelona (UAB), Barcelona, Spain; 2 Department of Cardiac Surgery, University Hospital Vall d’Hebron, Autonomous University of Barcelona (UAB), Barcelona, Spain; 3 Gene & Cell and Therapy Laboratory VHIR, UAB, Barcelona, Spain; University of Colorado Denver, UNITED STATES

## Abstract

**Background:**

The diseased human myocardium is highly susceptible to ischemia/reoxygenation (I/R)-induced injury but its response to protective interventions such as ischemic preconditioning (IPreC) is unclear. Cardiac and other pre-existing clinical conditions as well as previous or ongoing medical treatment may influence the myocardial response to I/R injury and protection. This study investigated the effect of both on myocardial susceptibility to I/R-induced injury and the protective effects of IPreC.

**Methods and results:**

Atrial myocardium from cardiac surgery patients (n = 300) was assigned to one of three groups: aerobic control, I/R alone, and IPreC. Lactate dehydrogenase leakage, as a marker of cell injury, and cell viability were measured. The basal redox status was determined in samples from 90 patients.

The response to I/R varied widely. Myocardium from patients with aortic valve disease was the most susceptible to injury whereas myocardium from dyslipidemia patients was the least susceptible. Tissue from females was better protected than tissue from males. Myocardium from patients with mitral valve disease was the least responsive to IPreC. The basal redox status was altered in the myocardium from patients with mitral and aortic valve disease.

**Conclusions:**

The response of the myocardium to I/R and IPreC is highly variable and influenced by the underlying cardiac pathology, dyslipidemia, sex, and the basal redox status. These results should be taken into account in the design of future clinical studies on the prevention of I/R injury and protection.

## Introduction

Cardiovascular diseases are the most prevalent health problem worldwide and the major cause of morbidity and mortality [1]. According to the WHO, in 2012 ischemic heart diseases caused 17.5 million deaths. Prolonged ischemia, such as occurs during myocardial infarction, causes lactate accumulation, alterations in ion-transport mechanisms, and eventually cardiomyocyte death through apoptosis and necrosis. However, oxygen restoration during reperfusion creates a large burst of reactive oxygen species production and calcium overload [2], causing additional myocardial damage. In fact, ischemic/reperfusion (I/R) injury is one of the main risk factors during heart surgery [3–5], especially in older patients and those with underlying comorbidities (e.g., diabetes) [6]. Over the last three decades, major efforts have been aimed at understanding the mechanisms of I/R-induced injury and developing strategies to protect the human myocardium. Nonetheless, much remains to be learned.

One of the main mechanisms of myocardial injury during I/R is oxidative and nitrosative stress, in which there is an imbalance between oxidant and antioxidant species that favors the former [7]. However, whether the basal redox status prior to the ischemic episode plays a significant role in I/R-induced myocardial injury is unknown. The main objectives of the present study were: (1) to determine myocardial susceptibility to I/R injury and the protective capacity of IPreC using myocardium obtained from patients undergoing elective cardiac surgery; (2) to investigate the effect of cardiac and other comorbidities and of medical treatment on I/R-induced injury and IPreC, and (3) to examine the role of the basal redox status in both these processes.

## Methods

### Study population

The study was approved by the Ethics Committee of Vall d'Hebron University Hospital (ID-RTF065) and conducted according to the principles expressed in the Helsinki Declaration. Written informed consent was obtained from each patient. The right atrial appendage was obtained from 300 patients undergoing elective cardiac surgery who were prospectively recruited without any exclusion criteria. Because of physical limitations, all patients had a sedentary life style before surgery. Demographic data, the presence of cardiovascular risk factors, and the medical treatment history of each patient were recorded.

### Human tissue collection and study groups

The right atrial appendages were collected in Krebs Henseleit Hepes (KHH) buffer containing (in mM): 118 NaCl, 4.8 KCl, 327.2 NaHCO, 1.2 MgCl_2_, 1 KH_2_PO_4_, 20 HEPES (Sigma, St. Louis, MO), pH 7.4 at 4–5°C. Briefly, the appendage was mounted epicardial surface face down onto a ground-glass plate and then sliced using surgical skin-graft blades (Swann-Morton, UK) to a thickness of 300–500 μm. The tissues slices (weight 30–50 mg) were kept moist throughout the procedure. Muscle pieces immediately frozen at −80°C were later used for the determination of basal redox status. The remaining muscle pieces were placed into a shaking water bath at 37°C and equilibrated for 30–40 min at 37°C in oxygenated buffer KHH containing 10 mM glucose and 1.25 mM CaCl_2_, pH 7.4.

**Fig 1:**
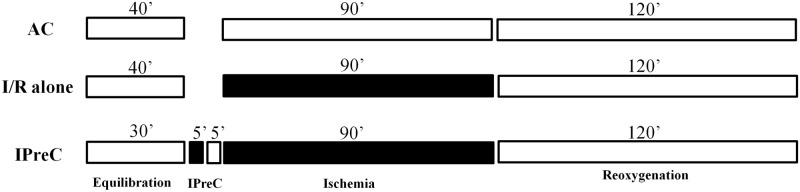
Experimental protocol. Tissues in all groups were equilibrated for 30–40 min at 37°C in aerobic conditions. Muscle tissues were either maintained under aerobic conditions (AC) for the entire experimental period or subjected to 90 min of ischemia followed by 120 min of ischemia/reoxygenation (I/R alone) or preconditioned (IPreC) with 5 min of ischemia and 5 min of reoxygenation.

The samples were then allocated to one of the following groups (Fig 1): (1) aerobic control (AC), in which the samples were incubated for 210 min in oxygenated KHH buffer (pH 7.4); (2) simulated ischemia/reoxygenation (I/R alone), induced by bubbling the incubation medium with 95% N_2_−5% CO_2_ (pH 6.8–7.0) and replacing d-glucose with 2-deoxy-d-glucose (Sigma, St. Louis, MO), as previously described [8]; and (3) IPreC, induced by 5 min of ischemia followed by 5 min of reoxygenation, applied before 90 min of ischemia and followed by 120 min of reoxygenation. In a previous study, we demonstrated that this protocol confers the greatest protection under laboratory conditions [9].

### Assessment of tissue injury and tissue viability

Tissue injury was assessed by the release of lactate dehydrogenase (LDH), which converts pyruvate to lactate, into the incubation medium at the end of the 120-min reoxygenation period. The absorbance was measured at a wavelength of 340 nm using a MultiSkan FC spectrometer (Thermo Fisher Scientific, Waltham, MA). The results were expressed as arbitrary units (AU)/g tissue wet wt.

The viability of the muscle tissue was assessed at the end of the 120-min reoxygenation period by the reduction of 3-(4,5-dimethyl thiazol-2-yl)-2,5-diphenyl tetrazolium bromide (MTT) (Sigma, St. Louis, MO) to a blue formazan product, the absorbance of which was measured at 550 nm using a MultiSkan FC spectrometer (Thermo Fisher Scientific, Waltham, MA). The results were expressed as AU/g wet wt.

### Protein extraction from myocardial tissues

Basal myocardial antioxidant capacity and oxidative and nitrosative stress were assessed in 90 myocardial tissue samples taken from unselected patients. After the samples were pulverized in liquid N_2_, they were transferred to phosphate buffer saline solution, pH 7.4 (15% v/w) (Sigma, St. Louis, MO) and sonicated. After 30 min on ice, the homogenates were centrifuged at 17000*g* for 15 min at 4°C. The supernatants were collected and used for the analyses. Protein amounts were quantified using the DC^™^ protein assay (Bio-Rad, Hercules, CA).

### Assessment of total antioxidant capacity (TAC)

The total antioxidant capacity was assessed using the Oxiselect^™^ assay kit (Cell Biolabs, San Diego, CA). The copper ion reagent (Cu^2+^) or a known concentration of uric acid was added to the samples. Upon reduction, Cu^2+^ reacts with a chromogenic reagent that produces an orange colored product. The absorbance of this product at 490 nm was measured using a MultiSkan FC spectrometer (Thermo Fisher Scientific, Waltham, MA). The results were expressed as mM of uric acid/mg protein.

### Manganese superoxide dismutase (MnSOD or SOD_2_) and catalase expression

Western blot experiments were conducted to measure MnSOD or SOD_2_ and catalase expression. Tissue sample were homogenized in lysis buffer (2% SDS, 10% glycerol, either 62.5 mM Trizma base, pH 6.8, or Tris HCl, protease, and phosphatase inhibitor). For western blot analysis, 80 μg of MnSOD and catalase proteins were separated electrophoretically on a 4–20% polyacrylamide gel and then transferred onto a nitrocellulose membrane using a semi-dry electric transfer device (Bio-Rad Laboratories, Hercules, CA). The membranes were then blocked in 5% nonfat dry milk in Tris-buffered saline and incubated with an anti-catalase or anti-MnSOD rabbit polyclonal antibody (EMD Millipore Corporation, San Diego, CA and Sigma, St. Louis, MO, respectively). Incubation with an anti-actinin goat polyclonal antibody served as the normalization control. The secondary antibodies were goat anti-rabbit (Sigma) and donkey anti-goat (Abcam, Cambridge, UK) conjugated with horseradish peroxidase. The bands were visualized using Clarity^™^ Western ECL (Bio-Rad Laboratories, Hercules, CA); the images were captured on a luminescent image analyzer LAS-3000 (FujiFilm Life Science, Stamford, CT). Individual MnSOD and catalase bands were quantified by densitometric scanning using the Image J program. The results were expressed as arbitrary densitometric units (ADU).

### Determination of superoxide anion production (O_2_^.−^)

The generation of O_2_^.−^ was determined using 2,3-dimethoxy-1,4-naphtoquinone (DMNQ), which is a redox-cycling agent that induces intracellular O_2_^.−^ formation. The reaction mixture, containing (in mM) 50 potassium phosphate, 1 ethylene diaminetetraacetic acid (EDTA), and 1 DMNQ (Sigma, St. Louis, MO), was incubated with 10 μl of tissue homogenate at 37°C for 3 h. Thereafter, native coelenterazine (Sigma, St. Louis, MO), which emits light from enzyme-independent oxidation in the presence of O_2_^.−^ and peroxynitrite, was added at a concentration of 100 μM. The emitted luminescence was measured with Gene 5 (Biotek, Winooski, VT). The results were expressed as luminescence units (LU)/μg protein.

### Determination of thiobarbituric-acid-reactive substances (TBARS)

Malondialdehyde (MDA) formation was used as an indicator of TBARS production. A mixture containing tissue homogenate, 0.5% butyl hydroxy toluene, 0.14 mM ethylene-diamine-tetra-acetic acid, 40% trichloro-acetic acid, and 0.67% thiobarbituric acid (TBA) was boiled at 100°C for 10 min to start the reaction. TBA reacts with MDA to yield a pink product. Aqueous and organic phases were separated by the addition of acetic glacial acid and chloroform followed by centrifugation at 1700*g* for 30 min. The absorbance of the organic phase was measured at 540 nm. The MDA concentration was calculated from a standard curve of 1,1,3,3-tetraethoxypropane. The results were expressed as μM TBARS/mg protein.

### Determination of nitric oxide (NO) bioavailability

The bioavailability of NO was assessed using a colorimetric assay kit (Cayman, Ann Arbor, MI) performed according to the manufacturer’s instructions. The samples were incubated with nitrate reductase, which converts nitrates to nitrites. The latter react with the Griess reagent to yield a colored azo dye product, the absorbance of which was measured at 540 nm using a MultiSkan FC spectrometer (Thermo Fisher Scientific Waltham, MA). The results were expressed as μM NO/mg protein.

### Determination of 3-nitrotyrosine

The OxySelect^™^ Elisa kit (Cell Biolabs, San Diego, CA) was used to measure 3-nitrotyrosine, a product of tyrosine nitration by peroxynitrite, production. The assay was carried out according to the manufacturer’s instructions. The results were expressed as nM 3-nitrotyrosine/mg protein.

### Statistical analysis

The results were expressed as the mean ± SEM. ANOVA and the Mann-Whitney test were used to compare the means between groups. Linear regression and logistic binary regression were used to compare the influence of the associated comorbidities and medical treatment. The redox indexes were analyzed using the Kruskal Wallis test, followed by a post-hoc Dunn’s test when indicated. Statistical analyses were performed using the SPSS 20 and Graph Pad Prism 6. A P value < 0.05 was considered to indicate statistical significance.

## Results

Table 1 summarizes the demographic data from the donor patients (n = 300).

**Table 1:** Demographic data of study patients.

Variable		N = 300 (ratio in %)
Sex		
	Female	94(31)
	Male	206(69)
Age (years)		65±12
Obesity		118(39)
Diabetes		
	Insulin-dependent	29(10)
	Non insulin-dependent	66(22)
Dyslipidemia		161(54)
Hypertension		207(69)
Coronary artery disease		123(41)
Aneurysm of ascending aorta		43(14)
Interatrial communication (ostium secundum)		3(1)
Left ventricular ejection fraction		
≥40		278(93)
<40		22(7)
Atrial fibrillation		
	Paroxystic	17(6)
	Permanent	27(9)
Aortic valve disease		175(58)
Mitral valve disease		46(15)
Tricuspid valve disease		10(3)

### Susceptibility of the human myocardium to I/R-induced injury

**Fig 2:**
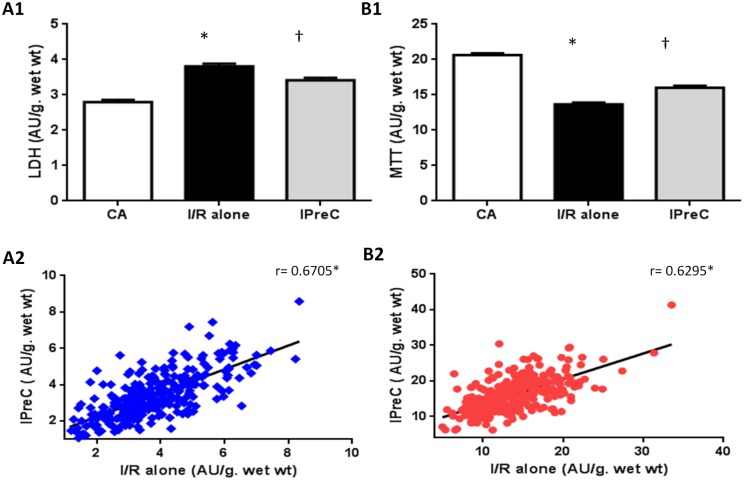
Lactate dehydrogenase (LDH) leakage (A1) and 3-(4,5-dimethyl thiazol-2-yl)-2,5diphenyl tetrazolium bromide (MTT) reduction (B1) and the correlation between IPreC and I/R alone for LDH release (A2) and MTT reduction (B2) of human myocardium muscles (n = 300 per group) subjected to 250 min of aerobic condictions (AC), 90 min of ischemia followed by 120 min of reoxygenation (I/R alone), or ischemic preconditioning (IPreC) induced by 5 min of ischemia followed by 5 min of reoxygenation prior to the 90 min of ischemia. The mean values are shown. *p < 0.05 vs AC group and †*p* < 0.05 vs I/R alone group.

Fig 2A1 and 2B1 shows the overall mean values for LDH and cell viability, respectively, for the three study groups (the mean ± SEM for all results are shown in Table A in S1 File). I/R alone induced a significant increase in LDH and a significant decrease in viability compared with the aerobic controls. In addition, IPreC significantly reduced the ischemic damage, evidenced by the reduction in LDH and the greater cell viability. Fig 2A2 and 2B2 show the wide-ranging response of human myocardium to I/R injury, as in some tissues there was only a small amount of LDH leakage and only a small decrease in viability, whereas in other tissues the LDH leakage and viability were substantially worse. As expected, the correlation between I/R injury and protection by IPreC was positive for LDH leakage and viability.

### Broad-spectrum response of human myocardium to IPreC

**Fig 3:**
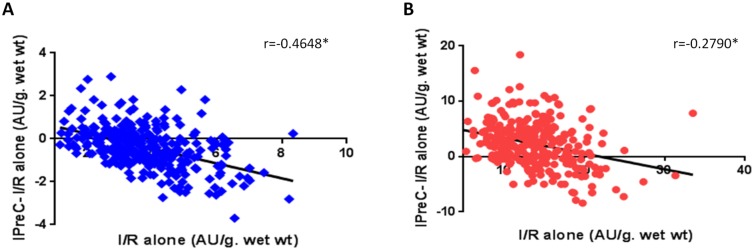
Correlation between IPreC-I/R alone and I/R alone values for LDH release (A) and MTT reduction (B) in human myocardial muscles (n = 300), subjected to 90 min of normothemic ischemia followed by 120 min of reoxygenation (I/R alone) and ischemic preconditioning (IPreC) induced by 5 min of ischemia followed by 5 min of reoxygenation prior to the 90 min of ischemia. *p < 0.05.

Fig 3A and 3B also demonstrates the wide-ranging response of human myocardium to protection by IPreC. Based on a comparison of the data from the IPreC and I/R alone groups, (negative LDH values and positive viability), the degree of protection was greater in the presence of more severe ischemic injury. The results also showed that muscle tissue with less ischemic injury either did not respond to preconditioning or was more susceptible to damage during IPreC. Thus, in 22% of the samples analyzed, the LDH and viability values of the IPreC group were worse than those of the I/R alone group. These findings suggest that protective interventions such as IPreC are unwarranted in patients with less myocardial damage.

### The role of cardiac pathologies, comorbidities, and medical treatment

The effects of underlying cardiac pathologies, comorbidities, and medical treatment on myocardial susceptibility to I/R-induced injury and the capacity for cardioprotection by IPreC were analyzed. Myocardium from patients with aortic valve disease was more susceptible to I/R injury than myocardium from patients with other cardiac pathologies, whereas myocardium from patients with dyslipidemia was the least susceptible (Table 2). Interestingly, comorbidities and medical treatment did not influence the susceptibility of the myocardium to ischemia (data shown in Tables B and C in S1 File). The protection conferred by IPreC was significantly better in myocardial tissues from females than from males. Among the cardiac diseases, myocardium from patients with mitral valve disease was the least responsive to IPreC protection (Table 3).

**Table 2:** Conditions affecting the susceptibility to ischemia/reoxygenation (I/R)-induced injury obtained by substracting the LDH values of the AC group from the I/R alone group for each individual patients and then compared with the mean value of the remainder study population.

Condition	N	Average LDH (AU/g. wet wt)	P–value*
With aortic valve disease	137	1.1	0.049
Without aortic valve disease	163	0.9	
With dyslipidemia	161	0.9	0.045
Without dyslipidemia	139	1.1	

*p values were calculated using the Mann Whitney test.

**Table 3:** Conditions affecting the capacity for cardioprotection by preconditioning (IPreC) obtained by substracting the LDH values of the I/R alone group from the IPreC group for each individual patients and then compared with the mean value of the remainder study population.

Condition	N	Average LDH (AU/g. wet wt)	P–value*
Female	94	-0.50	0.035
Male	206	-0.30	
With mitral valve disease	46	-0.12	0.038
Without mitral valve disease	254	-0.44	

*p values were calculated using the Mann Whitney test.

### Basal myocardial redox status and its influence on the response to ischemia and IPreC

Oxygen and nitrogen free radicals play important roles in ischemic injury, in the signal transduction mechanism that confers myocardial protection [10,11], and in the pathogenesis and development of cardiovascular diseases, particularly atherosclerosis [12]. To investigate whether the basal redox status influences the susceptibility of the human myocardium to I/R injury and whether IPreC has a protective effect, tissue levels of NO, MDA, 3-nitrotyrosine, TAC, O_2_^.−^, MnSOD, and catalase were measured in atrial muscle tissue from 90 unselected patients undergoing cardiac surgery. As seen in Table 4, myocardium from patients with aortic valve disease, shown in the above-described experiment to be more susceptible to I/R injury, had lower TAC and higher catalase values than myocardium from patients without aortic valve disease. Table 4 also shows that the MnSOD content was significantly higher in myocardium from patients with mitral valve disease, whose tissue was less responsive to IPreC-mediated protection, than in myocardium from patients without mitral valve pathologies.

**Table 4:** Indexes of basal redox status in human right atrial tissue (n = 90) from patients with conditions that affect myocardial susceptibilility to I/R-induced injury and the capacity for IPreC-mediated protection.

Index	With AVD	Without AVD	With DLP	Without DLP	Female	Male	With MVD	Without MVD
No (μM/mg prot)	52.25±5.3	45.68±5.4	48.69±4.8	51.39±6.5	48.12±4.6	52.58±6.8	49.53±8.1	49.81±4.4
MDA (μM/mg prot)	25.54±3.0	23.80±0.2	24.95±2.4	24.57±3.5	27.19±2.8	20.61±2.4	24.26±4.1	24.92±2.3
3-NT (nM/mg prot)	229.33±25.7	187.63±2.5	223.34±29.3	202.96±16.9	234.32±25.5	176±14.9	165.38±19.7	224.93±20.8
O_2_^.-^ (UL/μg prot)	27.51±1.3	29.84±17.6	28.21±1.4	28.20±2.0	28.00±1.4	28.67±1.8	30.23±2.5	27.73±1.3
TAC (mM Uric acid/μg prot)	*1*.*6±0*.08*	1.89±2.1	1.70±0.07	1.76±0.1	1.68±0.07	1.80±0.12	1.93±0.19	1.67±0.06
MnSOD (ADU)	0.89±0.04	0.88±0.1	0.87±0.04	0.91±0.04	0.85±0.03	0.96±0.04	*1*.*02±0*.07*	0.86±0.03
CAT (ADU)	*0*.*52±0*.03*	0.40±0.04	0.49±0.03	0.46±0.03	0.48±0.03	0.48±0.04	0.48±0.05	0.48±0.02

AVD = aortic valve disease, CAT = catalase, DLP = dyslipidemia, MDA = malondialdehyde, MnSOD = manganese superoxide dismutase, MVD = mitral valve disease, NO = nitric oxide, O_2_^.-^ = superoxide anion, TAC = total antioxidant capacity, 3-NT = nitrotyrosine.

* ***p*** < 0.05 values were calculated using the Mann Whitney test.

## Discussion

In this study, *in vitro* preparations of right atrial appendages from a very large cohort of patients undergoing elective heart surgery were examined with respect to I/R and IPreC. The results demonstrated that: (1) the response of the human myocardium to I/R is highly variable, with tissue from patients with aortic valve disease being more susceptible to injury than tissue from patients with other cardiac pathologies. By contrast, the myocardial tissue of patients with dyslipidemia was the least susceptible to I/R injury. (2) The response to IPreC was also highly variable. The myocardium from females was significantly better protected than that from males and, the myocardium from patients with mitral valve disease was less responsive to IPreC-mediated protection than tissues from patients without mitral valve disease. (3) The redox status of the myocardium under basal conditions (prior to ischemia) may be relevant to the degree of I/R-induced injury and to the response to IPreC in patients with heart valve pathologies.

Since, for obvious ethical reasons, the effect of I/R-induced injury on human myocardium cannot be investigated in clinical settings, we took advantage of a well characterized *in vitro* model [8] and were able to show large differences in the response of the human myocardium to I/R, ranging from slight to severe injury after 90 min of ischemia and 120 min of reoxygenation (Fig 2A2 and 2B2). The greater susceptibility of myocardial tissue from patients with aortic valve disease to I/R injury is consistent with previous reports showing that myocardial hypertrophy causes extensive I/R injury in animal experimental preparations [13,14] and in humans [15]. Our finding is of particular interest because the prevalence of left ventricular (LV) hypertrophy is 20–25% in the general population [16,17] and 50% in patients with previous myocardial infarction [18].

The increased extracellular collagen matrix of the hypertrophied myocardium together with its decreased capillary density could make patients with LV hypertrophy more susceptible to I/R injury. Furthermore, carbohydrate metabolism is altered in the hypertrophied left ventricle, which may contribute to an increased susceptibility to I/R injury, by causing disturbances in ion homeostasis. Earlier reports showed that the hypertrophied myocardium exhibits an accelerated loss of high-energy phosphate content and a greater accumulation of tissue lactate and hydrogen ions during ischemia as well as an accelerated calcium overload during early reperfusion [19,20]. It should be noted that the infarct size in patients with LV hypertrophy may be significantly overestimated when based on the determination of cardiac enzymes such as the peak and AUC plasma values of cardiac troponin I [21]. In the present study, the results were expressed based on the weight of the myocardial tissue, thus avoiding this source of potential error.

The effect of hypercholesterolemia, a risk factor for cardiovascular disease, on I/R-induced injury is controversial and the mechanisms involved are not well understood [22]. Both the type of experimental preparation used and the duration of the hypercholesterolemic diet in animal models may influence the degree of I/R-induced injury. Our results showed that the myocardium from patients with established dyslipidemia is less susceptible to injury. However, the majority of the patients in our study (95%) were taking HMG-CoA reductase inhibitors (statins), which in addition to reducing serum cholesterol exert pleiotropic effects that could have influenced the susceptibility of the myocardium to I/R injury [23]. Experimental studies have implicated an increase in oxidative stress and a diminished bioavailability in NO in the altered response to I/R injury in hypercholesterolemia [24]. Our assessment of oxidative and nitrosative stress basal status in human myocardium was unable to demonstrate its direct relationship with the degree of I/R injury in the myocardium of patients with hypercholesterolemia. Therefore, it cannot be argued that the diminished susceptibility to I/R injury of the myocardium from patients with dyslipidemia is due to changes in the basal redox status of the tissue.

The wide spectrum of responses of human myocardium to IPreC (Fig 3A and 3B), including the increased damage in 22% of IPreC-treated myocardial samples, calls into question the clinical utility of this intervention, at least when used indiscriminately. A univariate analysis of the various pathologies, comorbidities, and medical treatment revealed a better myocardial response to IPreC in female than in male patients, consistent with the well-established sex-related differences in the response to global injury in humans [25–27]. The mechanisms responsible for the higher degree of protection by IPreC in females have yet to be fully elucidated but seem to involve estrogens [28–30] and estrogen receptor-α [31]. Furthermore, endothelial nitric oxide synthase expression is higher in females than in males [32], suggesting that higher levels of NO play a role in the better cardioprotective potential of IPreC. Nonetheless, in our study neither the myocardial NO content nor the antioxidant status differed between females and males under basal conditions.

Another important finding of our study was the lower susceptibility of the myocardium from patients with mitral valve disease to IPreC protection. The increased pressure within the left atrium and pulmonary vasculature characteristic of mitral valve disease may lead to hypertrophy and dilation of the right heart [33], which would contribute to a reduced response to IPreC. The clinical importance of our findings lies in the fact that patients undergoing mitral valve surgery may be less responsive to cardioprotective therapies and at greater risk of perioperative myocardial injury, leading to heart failure. These patients may therefore require additional protective measures during heart surgery. The underlying mechanisms for the diminished response to IPreC in the presence of mitral valve disease are unknown. Cavalca et al. [34] reported an increase in the levels of oxidative stress markers in the serum of patients with mitral valve disease, but in our study, with the exception of the increased myocardial MnSOD levels, there were no significant differences in other indexes of tissue redox status under basal conditions (see below).

There is ample evidence in the literature of both the involvement of free radicals in the injury sustained during I/R and the benefit of interventions aimed at reducing their production. To the best of our knowledge, this is the first study to investigate whether the basal redox status of the myocardium influences the response to I/R-induced injury and the protective effect of IPreC. Our results demonstrated a role for basal redox status in the response to I/R injury and IPreC by the myocardium of patients with certain cardiac pathologies. Although the reason for the greater susceptibility to I/R injury of myocardium from patients with aortic valve disease is unclear, it may be that the increased myocardial catalase content reflects the body’s attempt to combat the excess oxidative stress. This would explain the decreased TAC levels, indicative of the consumption of antioxidants and thus of an increased susceptibility of the myocardium to I/R injury. The greater MnSOD content in the myocardium of patients with mitral valve disease, which is less responsive to protection by IPreC, is difficult to interpret. A possible explanation is that the resulting greater ability to mop up free radicals eliminates a critical trigger leading to the induction of IPreC. This conclusion is supported by a report in which IPreC was experimentally induced by oxygen radicals [35] but protection was attenuated by antioxidants [36]. Alternatively, the elevated MnSOD in the myocardial tissue of patients with mitral valve disease may be a natural response in line with the higher levels of oxidative stress markers in the serum of these patients [34]. It should be noted that in the present study redox status was determined in myocardial tissue, where it did not correlate with comorbidities, as opposed to plasma, in which elevated levels of oxidative and nitrosative stress markers have been determined in patients with diabetes [37], atherosclerosis, hypertension, and heart failure [37,38]. Whether anti-free radical therapies should be aimed at controlling the redox status in blood rather than in tissues remains to be examined in further investigations.

A major contribution of this study is its demonstration that the response to I/R and protective interventions such as IPreC by the diseased myocardium is neither uniform nor predictable, which may at least in part explain the failure to translate cardioprotective therapies into useful clinical treatments. Our results indicate the need for caution in the broad clinical use of IPreC and instead emphasize the importance of further studies, using relevant human disease models, to fully identify the molecular basis of I/R injury and the appropriate cardioprotective interventions in patients. These efforts will improve treatment approaches in the clinical setting.

A limitation of the present study is that, for ethical reasons, only right atrial tissue and not left ventricular myocardium could be obtained. However, we [39] and others [40] have demonstrated that the myocardium from the two cardiac chambers responds similarly to I/R and IPreC. Nonetheless, it remains possible that cardiac valve pathologies differentially affect the cardiac chambers and thereby influence the myocardial response to I/R and cardioprotective therapies. Another limitation was that the study was performed *in vitro* using isolated myocardial tissue; therefore, our results cannot necessarily be extrapolated to clinical conditions. Nonetheless, the laboratory model used in this study provides a safe, rapid, and inexpensive system for testing and refining protective interventions in human myocardium impaired by various comorbidities and/or exposed to medical treatment, before they are considered for clinical use.

## Conclusions

The response of the myocardium from patients with heart diseases to I/R and IPreC varies widely and may be influenced by specific cardiac pathologies and the sex of the patient. Our results do not support the broad clinical use of IPreC; rather, they emphasize the need for research into both the molecular basis of I/R-induced injury and the cardioprotective interventions in human myocardium that will lead to clinical success. Whether the basal redox status of the myocardium plays a role in the degree of I/R-induced injury and the response to IPreC remains to be determined. Our results are an important contribution to the design of future clinical studies on I/R injury and IPreC.

## Supporting information

S1 File Table A. Mean values for lactate dehydrogenase (LDH) and 3-(4,5-dimethyl thiazol-2-yl)-2,5diphenyl tetrazolium bromide (MTT) in human right atrial appendage (n = 300). Table B. Conditions affecting the susceptibility to ischemia/reperfusion (I/R)-induced injury. No statistically significant differences where observe between groups. Table C. Conditions affecting the capacity of protection by ischemic preconditioning. No statistically significant differences where observe between groups.
(DOCX)
