# Supplementary material for: Response of the human myocardium to ischemic injury and preconditioning: The role of cardiac and comorbid conditions, medical treatment, and basal redox status
Source: PLoS One. 2017 Apr 5;12(4):e0174588. doi: 10.1371/journal.pone.0174588 (PMC5381881; doi:10.1371/journal.pone.0174588)
Supplement: S1 File — Table A. Mean values for lactate dehydrogenase (LDH) and 3-(4,5-dimethyl thiazol-2-yl)-2,5diphenyl tetrazolium bromide (MTT) in human right atrial appendage (n = 300). Table B. Conditions affecting the susceptibility to ischemia/reperfusion (I/R)-induced injury. No statistically significant differences where observe between groups. Table C. Conditions affecting the capacity of protection by ischemic preconditioning. No statistically significant differences where observe between groups. (DOCX) [file pone.0174588.s001.docx]

**S1 Table A. Mean values for lactate dehydrogenase (LDH) and 3-(4,5-dimethyl thiazol-2-yl)-2,5-diphenyl tetrazolium bromide (MTT) in human right atrial appendage (n = 300).**

|  | **LDH** |  | **MTT** |  |
| --- | --- | --- | --- | --- |
|  | **Average(AU/g. wet wt)** | **P – value*** | **Average (AU/g. wet wt)** | **P – value*** |
| AC | 2.8 |  | 20.6 |  |
| I/R | 3.8 | 0.023 | 13.6 | 0.019 |
| IPreC | 3.4 | 0.040 | 16.0 | 0.045 |

AC= aerobic control group, I/R= ischemia/reoxigenation group, IPreC= ischemic preconditioning group.

**p* < 0.05, values was calculated using the Mann Whitney test.

**S1 Table B.** **Conditions affecting the susceptibility to ischemia/reperfusion (I/R)-induced injury. No statistically significant differences where observe between groups**

| **Condition** | **N** | **Average LDH (AU/g. wet wt)** | **P- value** |
| --- | --- | --- | --- |
| With AAA  Without AAA | 43  257 | 1.2  1.0 | 0.100 |
| With CAD  Without CAD | 123  177 | 0.9  1.1 | 0.066 |
| With AF  Without AF | 56  244 | 1.0  1.0 | 0.887 |
| With hypertension  Without hypertension | 207  93 | 0.96  1.10 | 0.274 |
| With diabetes  Without diabetes | 95  205 | 0.9  1.1 | 0.065 |
| With obesity  Without obesity | 118  182 | 1.0  1.0 | 0.658 |
| <45 years  >45 years | 65  235 | 1.1  0.9 | 0.153 |

AAA= Aneurysm of ascending aorta, CAD= Coronary artery disease, AF= Atrial fibrillation

**S1 Table C. Conditions affecting the capacity of protection by ischemic preconditioning. No statistically significant differences where observe between groups**

| **Condition** | **N** | **Average LDH (AU/g. wet wt)** | **P- value** |
| --- | --- | --- | --- |
| With AAA  Without AAA | 43  257 | -0.46  -0.37 | 0.808 |
| With CAD  Without CAD | 123  177 | -0.37  -0.40 | 0.897 |
| With AF  Without AF | 56  244 | -0.32  -0.41 | 0.174 |
| With hypertension  Without hypertension | 207  93 | -0.40  -0.45 | 0.642 |
| With diabetes  Without diabetes | 95  205 | -0.29  -0.43 | 0.283 |
| With obesity  Without obesity | 118  182 | -0.42  -0.37 | 0.350 |
| <45 years  >45 years | 65  235 | -0.36  -0.47 | 0.397 |

AAA= Aneurysm of ascending aorta, CAD= Coronary artery disease, AF= Atrial fibrillation
